# Streptococcal Toxic Shock Syndrome Caused by Streptococcus suis Serotype 2

**DOI:** 10.1371/journal.pmed.0030151

**Published:** 2006-04-11

**Authors:** Jiaqi Tang, Changjun Wang, Youjun Feng, Weizhong Yang, Huaidong Song, Zhihai Chen, Hongjie Yu, Xiuzhen Pan, Xiaojun Zhou, Huaru Wang, Bo Wu, Haili Wang, Huamei Zhao, Ying Lin, Jianhua Yue, Zhenqiang Wu, Xiaowei He, Feng Gao, Abdul Hamid Khan, Jian Wang, Guo-Ping Zhao, Yu Wang, Xiaoning Wang, Zhu Chen, George F Gao

**Affiliations:** 1Department of Epidemiology, Research Institute for Medicine of Nanjing Command, Nanjing, China; **2**Center for Molecular Immunology and State Key Laboratory of Microbial Resources, Institute of Microbiology, Chinese Academy of Sciences, Beijing, China; **3**Chinese Center for Disease Control and Prevention (China CDC), Beijing, China; **4**State Key Laboratory of Medical Genomics, Ruijin Hospital Affiliated to Medical School of Shanghai Jiao-Tong University, Shanghai, China; **5**Beijing Ditan Hospital, Beijing, China; **6**Department of Pathology, Jinling Hospital of Nanjing, Nanjing, China; **7**School of Biosciences and Bioengineering, South China University of Technology, Guangzhou, China; **8**Beijing Genomics Institute, Chinese Academy of Sciences, Beijing, China; **9**Chinese National Human Genome Center, Shanghai, China; **10**Graduate School, Chinese Academy of Sciences, Beijing, China; Free University of BrusselsBelgium

## Abstract

**Background:**

Streptococcus suis serotype 2 ( *S*. *suis* 2, SS2) is a major zoonotic pathogen that causes only sporadic cases of meningitis and sepsis in humans. Most if not all cases of Streptococcal toxic shock syndrome (STSS) that have been well-documented to date were associated with the non-SS2 group A streptococcus (GAS). However, a recent large-scale outbreak of SS2 in Sichuan Province, China, appeared to be caused by more invasive deep-tissue infection with STSS, characterized by acute high fever, vascular collapse, hypotension, shock, and multiple organ failure.

**Methods and Findings:**

We investigated this outbreak of SS2 infections in both human and pigs, which took place from July to August, 2005, through clinical observation and laboratory experiments. Clinical and pathological characterization of the human patients revealed the hallmarks of typical STSS, which to date had only been associated with GAS infection. Retrospectively, we found that this outbreak was very similar to an earlier outbreak in Jiangsu Province, China, in 1998. We isolated and analyzed 37 bacterial strains from human specimens and eight from pig specimens of the recent outbreak, as well as three human isolates and two pig isolates from the 1998 outbreak we had kept in our laboratory. The bacterial isolates were examined using light microscopy observation, pig infection experiments, multiplex-PCR assay, as well as restriction fragment length polymorphisms (RFLP) and multiple sequence alignment analyses. Multiple lines of evidence confirmed that highly virulent strains of SS2 were the causative agents of both outbreaks.

**Conclusions:**

We report, to our knowledge for the first time, two outbreaks of STSS caused by SS2, a non-GAS streptococcus. The 2005 outbreak was associated with 38 deaths out of 204 documented human cases; the 1998 outbreak with 14 deaths out of 25 reported human cases. Most of the fatal cases were characterized by STSS; some of them by meningitis or severe septicemia. The molecular mechanisms underlying these human STSS outbreaks in human beings remain unclear and an objective for further study.

## Abstract 2

Clinical description of a 2005 *S. suis* outbreak in China that affected over 200 individuals and initial characterization of the pathogenic isolates from affected pigs and humans.

## Introduction

Streptococcus suis is responsible for a variety of diseases in pigs, including meningitis, septicemia, arthritis, pneumonia, and even acute death, causing great economic loss in the pig industry worldwide every year [ 1, 2]. So far, 35 different serotypes of S. suis have been identified based on variation in the capsular antigens [ 2]. S. suis serotype 2 ( S. suis 2 or SS2) is the most frequently isolated serotype worldwide, and it comprises pathogenic, weakly pathogenic, and nonpathogenic strains [ 1– 4]. Various bacterial components such as extracellular and cell membrane–associated proteins have been suggested as virulence-associated factors, but their precise roles in the pathogenesis or virulence of *S*. *suis* have not been established [ 3, 4]. Currently, attempts to control the diseases caused by S. suis are still hampered by the lack of sufficient knowledge of pathogenesis mechanisms. Besides causing disease in pigs, S. suis 2 can also cause serious enzootic infections in humans, which are associated with septicemia, meningitis, and endocarditis [ 5]. There have been nearly 200 cases of S. suis 2 infection in humans worldwide since the first case of meningitis was recorded in Denmark in 1968 (China CDC, http://www.chinacdc.net.cn). Streptococcal toxic shock syndrome (STSS) refers to severe human invasive infection caused by *Streptococcus pyogenes (S*. *pyogenes),* a member of the group A streptococci (GAS) [ 6– 8] (Note, the abbreviation of STSS originally referred to staphylococcal toxic shock syndrome [TSS] and was borrowed later for TSS caused by S. pyogenes). Most invasive GAS disease occurs sporadically, but recent years have seen a rise of cases reported worldwide [ 9– 11]. GAS produces many toxins responsible for the clinical manifestations seen in infected patients. Some of them, labeled streptococcal pyrogenic exotoxins, have been characterized as superantigens [ 12, 13]. More recently, M protein, a constituent of the cell wall of *S. pyogenes,* has been found to be an important virulent factor and implicated in the pathological mechanism of STSS [ 14, 15].

STSS caused by non-GAS streptococci, including group B streptococci, group C streptococci, and group G streptococci, has not been well documented [ 16, 17]. Here we describe two recent outbreaks of *S*. *suis* 2, a non-GAS, in China and characterize the STSS they caused in human patients.

## Methods

### Clinical Observations

Faced with an unknown human infection outbreak in June 2005, in Ziyang County, Sichuan Province, China, we formed a team to investigate the event and to provide a comprehensive characterization of the patients. As we recognized the similarity to an earlier outbreak in 1998, we also analyzed retrospective data and analyzed stored samples from that outbreak. All work on autopsy samples (including the immunohistochemistry experiments described below) was carried out with written consent from relatives and approved by the Sichuan Provincial CDC ethics committee.

### Isolation and Identification of the Pathogen

To uncover the cause of the mysterious outbreak, blood, liver, and spleen samples were collected from the affected swine and humans. To isolate a possible bacterial pathogen, several selective media ( Protocol S1) [ 18] were utilized to propagate inoculums from these samples. Subsequently, Gram staining was performed according to the standard protocol [ 18], and the resultant slides were observed under an optical microscope. Candidate isolates were grown in Todd-Hewitt broth (THB, code CM189; Oxoid, Basingstoke, Hampshire, United Kingdom) for genomic DNA isolation, and plated on Columbia agar blood base (code CM331; Oxoid) containing 6% (vol/vol) sheep blood to differentiate their haemolytic types [ 18].

### Light and Electron Microscopy of Pathological Sections

To study pathological changes of the patients with *S*. *suis* 2–caused STSS, we used light microscopy for initial observations. Liver and spleen tissues from the autopsy specimen were selected to prepare sections for further transmission electron microscopy (TEM) observation ( Protocol S1).

### Immunohistochemistry Experiments

To examine details of the pathological changes and detect specific antigens in the infected tissues, immunohistochemistry was applied to formalin-fixed paraffin-embedded liver sections ( Protocol S1). Primary antibodies (anti– S. suis 2 serum derived from the recovered pig infected by S. suis 2) and normal pig serum were used at a dilution of 1:50. The Histo-stain Plus Kit (Zymed Laboratories, South San Francisco, United States) was used for detection. Sections were briefly counterstained with haematoxylin.

### Pig Infection Experiments

To test the pathogenicity of the bacterium agent, infection experiments were performed in pigs [ 19, 20] ( Protocol S1). The challenged piglets were monitored for clinical signs of the disease every 3 h. Bacterial pathogens were recovered from those piglets that showed marked signs of illness or had died 30 h post-infection. All experiments were conducted in a biosafety level 3 (BSL-3) facility and approved by the local Sichuan Provincial CDC ethics committee.

### Mitogenicity Activity Test to Search for Superantigens

To determine the mechanism by which potential superantigens might be produced by S. suis 2, mitogenicity activity was tested in human PBMCs [ 21, 22] ( Protocol S1).

### Qualitative PCR Assay

Routine genomic DNA preparations from all the isolates were done as described [ 23]. Multiplex-PCR (multi-PCR) assays were developed as described [ 24– 26]. Six pairs of primers specific for the target genes (including *16S rDNA,* glutamate dehydrogenase *[gdh],* extracellular protein factor *[epf], mrp, suilysin,* and *cps-2J*) were designed (listed in Table S1). Multi-PCR assays were performed using a PTC-225 thermocycler (MJ Research, Waltham, Massachusetts, United States), in a total volume of 50 μl. Genomic DNA from the R 735 strain of *S*. *suis* 2, provided by Marcelo Gottschalk in Canada, was used as a positive control template.

### RFLP Analysis of the Isolates

To test for potential genotypic differences among isolates from both outbreaks, we carried out chromosomal *Eco*RI digestion and RFLP analysis as previously described [ 23].

### Amplification and Sequencing of the Target Genes

General PCR was performed to amplify the full-length target genes encoding 16S rRNA and several virulence associated factors for further sequence analysis. A series of primers were designed to amplify the genes encoding 16S rDNA (one fragment), *gdh* (one fragment), suilysin (one fragment), fibronectin binding protein *(fbp), epf* (five fragments), muramidase release protein *(mrp)* (eight fragments), and a gene cluster involved in CPS2 synthesis (12 putative structural genes) (22 fragments) for sequencing by ABI 3730 DNA sequencer (Perkin-Elmer Applied Biosystems, Wellesley, Massachusetts, United States). Data were assembled and analyzed using Vector NTI Suite 8.0 software (Invitrogen, Carlsbad, California, United States). Phylogenic trees were constructed with MEGA 3.0 Software [ 27].

## Results

### Clinical Features of the Affected Patients

From June to August, 2005, an outbreak of severe invasive infection in humans and pigs occurred in Ziyang County, Sichuan Province, China, and attracted worldwide news coverage. A total number of 204 human cases, with 38 deaths ( Table 1) were recorded (China CDC, http://www.chinacdc.net.cn). The patients consisted of 198 farmers, five butchers, and one veterinarian, and most of them were found to have skin cuts on their hands and/or feet. Furthermore, they all had a history of direct contact with ill or dead pigs before they developed symptoms. No cases of human-to-human transmission were observed in this outbreak ( Table S2). The outbreak was immediately recognized as a zoonotic disease, because almost all the patients had been in contact with pigs, and there was a concurrent disease outbreak among the local pig population, with more than 640 pig fatalities. The disease was clearly serious and associated with death in a substantial fraction of patients ( Table 2). In the fatal human cases, the disease started with acute illness, malaise, fever, headache, diarrhea, rapidly developing hyperpyrexia, hypotension, and a decline of pulse pressure ( Table 3). Erythematous blanching rash was very obvious on the distal part of the extremities, including blood spots and petechia ( Table 3). All of these symptoms are indicative of possible TSS. Coma was seen in severe cases ( Table 3). It was common for not only the white blood cell count to be elevated ( Table 4), but also for the neutrophil cell count (data not shown). Ultimately, some cases progressed to multisystem dysfunction, such as acute respiratory distress syndrome (ARDS), liver failure, heart failure, disseminated intravascular coagulation (DIC), and acute renal failure ( Table 3). These symptoms and the hematological changes were in accordance with the clinical parameters of STSS caused by GAS [ 28, 29]. All but one of the 38 fatal cases had STSS symptoms. One death was due to meningitis. Patients with meningitis alone had fever, chills, headache, vomiting, and meningism. Retrospectively, the clinical features were extremely reminiscent of those associated with the earlier outbreak in 1998 [ 30, 31].

**Table 1:**
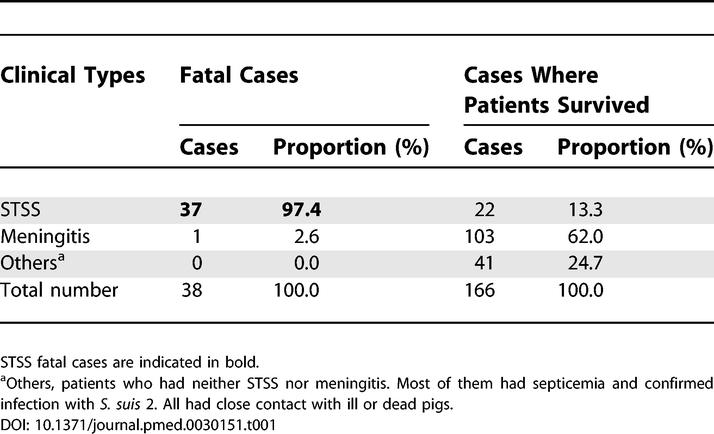
General Description of Human Cases from the 2005 Outbreak of SS2 Infections

**Table 2:**
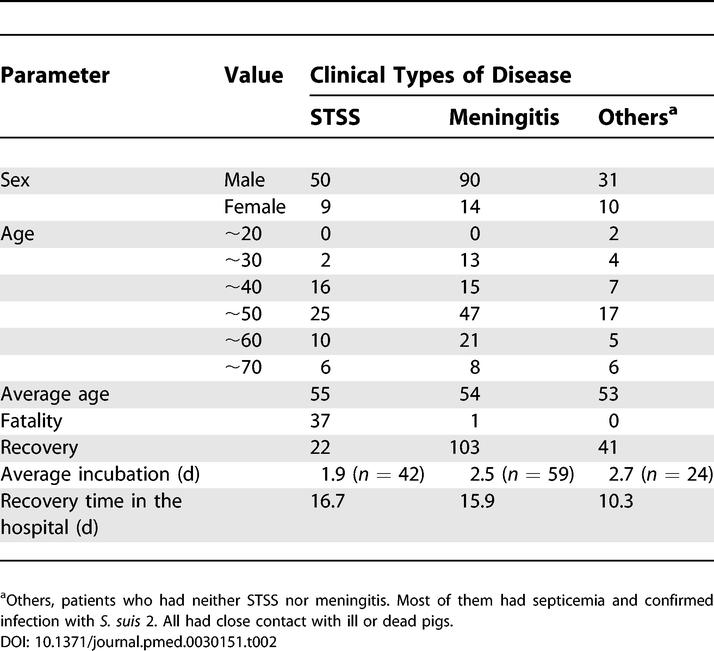
Details of Human Cases from the 2005 Outbreak of SS2 Infections

**Table 3:**
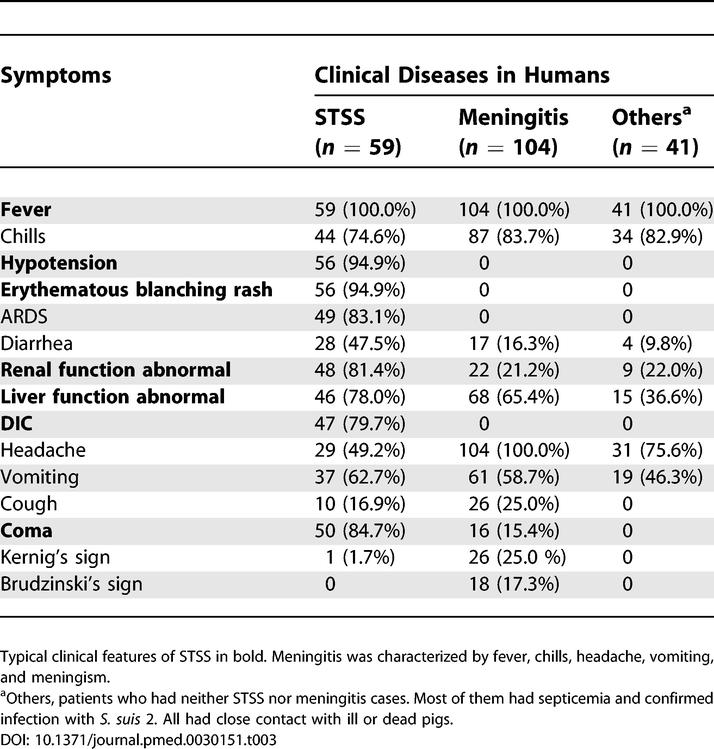
Clinical Features of Human Cases in the 2005 Outbreak of SS2 Infections

**Table 4:**
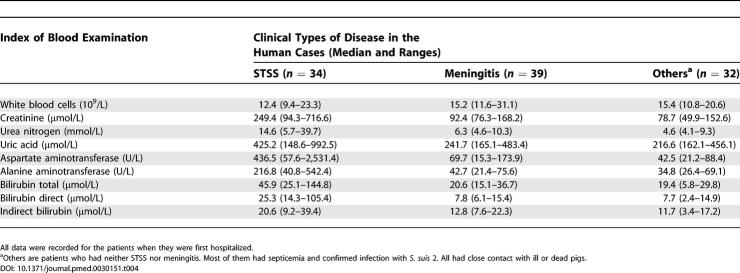
Haematology and Biochemistry of Different Clinical Diseases in Some of the Humans from the 2005 Outbreak of SS2 Infections

### Identification and Characterization of the Pathogen

**Figure 1:**
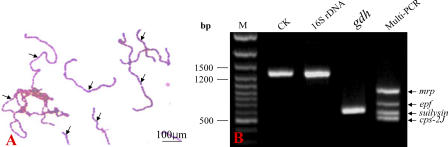
Detection of the Pathogenic SS2 and Identification of Its Specific Genes (A) Light microscopy image of the isolates cultured from autopsy specimens. GP ^+^ cocci (pointed to with black arrows) are arranged in various short chains (×100). (B) Qualitative PCR detection of isolates from the liver of fatal human cases with a set of primers specific for *S*. *suis* 2. M: 100bp DNA marker (Fermentas, Vilnius, Lithuania). CK: 16S rDNA PCR product from the R 735 standard strain of S. suis 2. Multi-PCR: performed with a set of unique primers specific for *mrp, epf, suilysin,* and *cps-2J,* respectively.

Based on the clinical features, we suspected that the patients had bacterial TSS, and we sought to identify the pathogenic bacteria. After two days of selective cultivation, we observed enriched growth of isolates from tissue samples of affected swine and human. On the agar plates with sheep blood, the bacteria showed α-haemolysis typical of *S*. *suis,* with the slightly gray or semitransparent, wet, and ropy colonies surrounded by haemolytic circles of 2–3 μm in diameter [ 18]. A subsequent Gram's stain experiment confirmed that all isolates were Gram-positive (GP ^+P^) cocci visible as short chains under the light microscope ( Figure 1A), suggesting that they may belong to the genus *Streptococcus*. Multi-PCR detection combined with direct DNA sequence analysis ( Protocol S1) was used to further determine the isolates' identity. The results identified the isolates as S. suis 2, with five putative virulence-factor genes, including *gdh, mrp, epf, suilysin,* and *cps-2J* ( Figure 1B). In total, we isolated and analyzed 37 strains from human specimens and eight from pig specimens. We also performed an analysis of three human isolates and two pig isolates from the 1998 outbreak and found exactly the same results.

### Pathological Changes in Dying Patients with STSS

**Figure 2:**
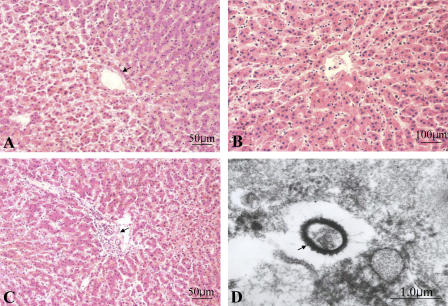
Microscopic Characterization of Sectioned Liver Tissue from Patients Who Had Died (A) Light image of a liver tissue section (×100). The central vein is indicated with an arrow. (B) Light image of a liver tissue section (×200). (C) The convergent zone is indicated with an arrow (×100). (D) TEM image of a liver tissue section (×20,000). A bacterium found in the tissue is highlighted with an arrow.

**Figure 3:**
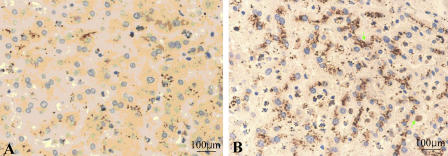
Immunohistochemical Analysis of Liver Tissue Sections from Dead Patients Incubated with Normal and S. suis 2-Infected Swine Serum (A) Staining with normal swine serum detected no S. suis 2 antigen. (B) Staining with the serum from the infected swine indicated the presence of S. suis 2 antigen.

To examine the pathological changes in patients dying of the presumed STSS caused by this pathogen, light microscopy was used, and more detailed pathological information was obtained using TEM ( Protocol S1). Both liver and spleen exhibited tissue autolysis. However, hepatic lobule structure and the tandem arrangement of the hepatic plates were relatively normal ( Figure 2A). Defuse degeneration of the hepatic cells was also seen ( Figure 2A), particularly in the areas surrounding the central vein ( Figure 2B). Hepatic cell necrosis was also observed in this area ( Figure 2B). The liver sinuses were slightly dilated due to proliferation of sinus histiocytes ( Figure 2B), while the hepatic cells around the convergent zone appeared unchanged ( Figure 2C). Although the structure of the spleen was also affected, both red and white pulps were clearly visible, with some reduction of the white pulps. Neither the liver nor the spleen showed obvious signs of inflammation. The tissue autolysis affected the cellular structures of the liver and spleen, but pathogenic bacteria were visible inside the Kupffer cells of the liver ( Figure 2D) and the macrophages of the spleen. The bacteria had oval shapes, 600–900 nm in length, and 280–580 nm in diameter, and were surrounded by glycocalyx ( Figure 2D). We performed immunohistochemistry on paraffin-embedded sections from the autopsy specimen, using sera from pigs infected with S. suis 2 and healthy pigs as negative control, respectively ( Figure 3). The autopsy specimen contained showed clearly visible positive brown particles in hepatic cells and Kupffer cells near the central vein ( Figure 3B), which were absent the negative control sample ( Figure 3A). All of these results suggested some crucial physical and pathological links between the STSS of the patients and S. suis 2.

### Pathogenesis Assessment of S. suis 2

To test the virulence of the field isolates, we infected SPF mini-piglets (Guizhou line) with S. suis ZYH13 that we had isolated from one of the fatal human cases of STSS. Two of the piglets were intravenously injected and both died within 27 h. Two others were intranasally inoculated, and they developed signs of severe illness (lamping, shivering high temperature, CNS failure, and respiratory failure) within 28 h, and both died at day 10 post-infection. In contrast, four piglets infected intravenously or intranasally (two each) with S. suis 68, a weakly virulent strain isolated from a healthy pig, all survived with only minor symptoms. Moreover, many GP ^+P^ cocci recovered from the tissue and blood of the infected pigs that had subsequently died were confirmed to be S. suis 2 by optical microscopy and qualitative PCR detection assays. Thus, the animal infection experiments confirmed that these isolates are highly pathogenic strains and supported the conclusion that an invasive strain of S. suis 2 caused the STSS symptoms associated with the human outbreaks.

### Superantigen Test and Genotyping of S. suis 2

**Figure 4:**
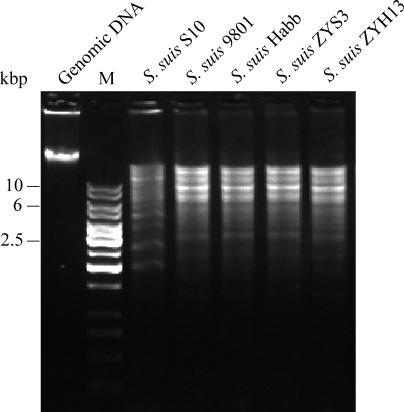
RFLP Analysis of Different S. suis 2 Isolates S. suis S10: a highly virulent strain from China; *S*. *suis* 9801: swine isolate from Jiangsu Province in 1998; S. suis Habb: human isolate from Jiangsu Province in 1998; S. suis ZYS3: swine isolate from Sichuan Province in 2005; S. suis ZYH13: human isolate from Sichuan Province in 2005; M: 1 kb DNA Ladder (MBI Ferments, Gdansk, Poland).

We failed to identify so-called superantigens in our isolates (unpublished data), suggesting that the molecular mechanisms by which S. suis 2 causes STSS might be different from those of GAS [ 12, 13]. RFLP analysis of the isolates from both outbreaks confirmed that they share a common genotype that is different from that of S. suis S10, an international strain of high virulence, and may constitute an unique strain of S. suis 2 ( Figure 4). Whole-genome sequencing of our isolates of S. suis 2 currently under way will help delineate the sequence differences with *S*. *suis* S10 and might suggest how the isolates cause STSS.

### Examination of Virulence-Associated Factors

**Figure 5:**
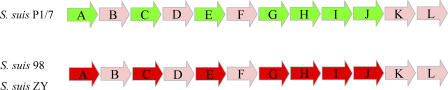
Comparison of the *cps2 G*ene Clusters Involved in CPS-2 Synthesis Exactly matched genes (5/12) include *cps2B, cps2D, cps2F*, *cps2K,* and *cps2L* and are shown in pink. The seven other genes (which did not exactly match) include *cps2A cps2C, cps2E, cps2G, cps2H, cps2I,* and *cps2J* and were highlighted with green in S. suis P1/7 and with red in both S. suis 98 and S. suis ZY. Amino acid abbreviations used are: A, alanine; C, cysteine; E, glutamic acid; F, phenyalanine; G, glycine; I, isoleucine; K, lysine; L, leucine; M, methionine; Q, glutamine; R, arginine; S, serine; V, valine; W, tryptophan; Y, tyrosine.

To further characterize the molecular features of the isolates from the two outbreaks, and to compare them with each other and with other unrelated isolates, we sequenced a number of genes and constructed phylogenic trees. The genes we analyzed included *16S rDNA, gdh,* structural gene clusters involved in capsule polysaccharide (CPS) synthesis (a total of 12 genes from *cps-2A* to *cps-2L*), *mrp, epf, suilysin,* and *fbp*. For the CPS gene clusters, both the current and 1998 isolates gave rise to a different pattern from the pattern for *S*. *suis* P1/7, an international highly pathogenic strain ( Figure 5), indicating specific characteristics shared by the S. suis 2 isolates from China. Five of 12 structural genes, including *cps2B, cps2D, cps2F, cps2K,* and *cps2L* exactly matched those of S. suis P1/7. In contrast, seven structural genes showed some point variations. The divergent genes included *cps2A* (G163A, F273I, and F302L), *cps2C* (I160V and Q195K), *cps2E* (G190E, W264L, and S281C), *cps2G* (F183Y), *cps2H* (M268I and K300E), *cps2I* (K145M), and *cps2J* (Q175K, R201S, S213G, L214I, and Y223K).

**Figure 6:**
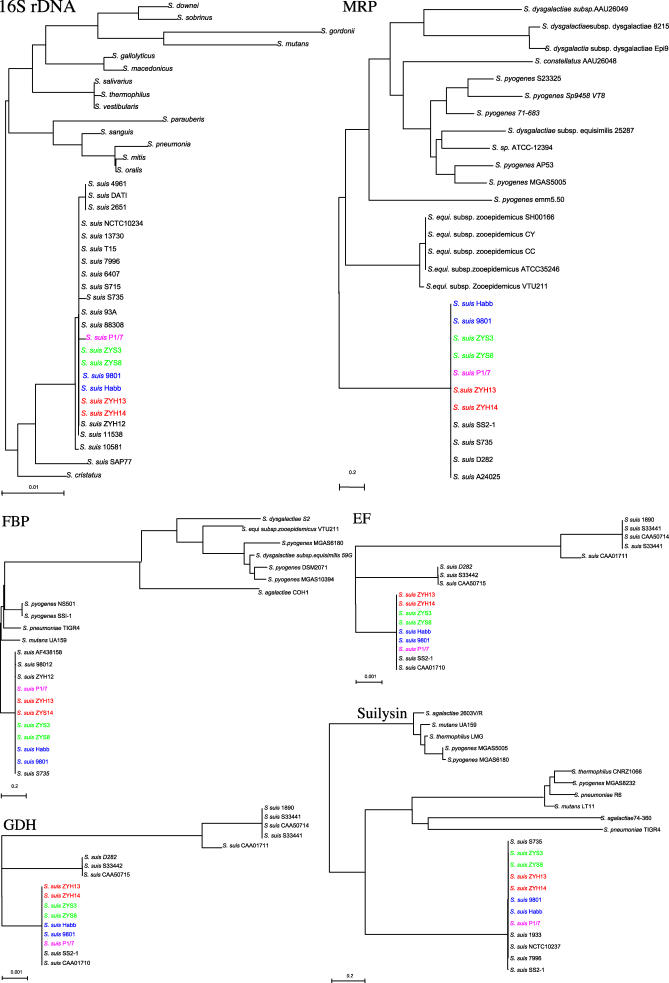
Phylogenetic Trees of Six Representative Isolates Based on Comparison of 16S rDNA and Five Putative Virulence-Associated-Factor Genes with Known Sequences Swine isolates from Sichuan ( S. suis ZYS3 and S. suis ZYS8) labeled in green, human isolates ( S. suis ZYH13 and S. suis ZYH14) from Sichuan labeled in red, Jiangsu isolates from 1998 ( S. suis 9801 and S. suis Habb) labeled in blue, and the standard highly virulent strain S. suis P1/7 labeled in pink. All representative strains from other streptococcus species or isolates of S. suis 2 are as indicated in the tree.

The sequencing results of the partial 16S rDNA and GDH (which has a possible role in pathogenesis [ 25, 32]) further confirmed the identity of S. suis 2 of the isolates ( Figure 6). All other sequencing data also indicated that the isolates represented a virulent strain of *S*. *suis* 2. Particularly EF, the derivative of EF*, which lacks the repeated amino acid units in the C-terminus and was initially described by Smith et al. [ 33] and is indicative of high virulence, was found in all our isolates. Phylogenic trees ( Figure 6) showed that our four isolates cluster together for one of the six genes we examined, which suggests that the same pathogenic bacteria might be involved in both outbreaks. Furthermore, the isolates shared five putative pathogenesis islands with a universal highly virulent S. suis P1/7. Taken together, our results indicate that the same or closely related pathogenic bacteria belonging to a highly virulent strain of *S*. *suis* 2 were responsible for two recent outbreaks and can cause STSS in humans.

## Discussion

*S*. *suis* 2 is known worldwide for causing sporadic meningitis and septicemia in pigs and humans who acquire the infection through skin wounds [ 1, 2, 5]. The first case of human meningitis caused by *S*. *suis* 2 in Denmark was reported in 1968. Prior to this study, we are aware of 198 cases of human S. suis infection worldwide, most of them caused by *S*. *suis* 2 and associated with clinical symptoms of meningitis or septicemia. However, this is to our knowledge the first detailed report in an international journal of STSS caused by S. suis 2 in a large-scale outbreak.

We describe an S. suis 2–caused STSS in a substantial number of human patients and a concurrent outbreak in pigs in Sichuan Province, China, and include some data from a similar outbreak in 1998 in Jiangsu Province, China. Our diagnosis was based on the following criteria, which might be useful for future identification of similar outbreaks: sudden onset of high fever; hypotension; blood spots and petechia; clear erythematous blanching rash; and dysfunction of multiple organs. Initially we speculated that there might be some special molecular markers for those isolates compared with standard strains. However, nearly all the candidate pathogenesis islands or virulence markers of our isolates, i.e., GDH, MRP, EF, Suilysin, and FBP [ 25, 32– 38] matched exactly those of S. suis P1/7 ( Figure 6), suggesting that the genes are conserved. Our data indicate that highly virulent strains of *S*. *suis* 2–caused human STSS in two recent large-scale outbreaks. However, the exact mechanism by which S. suis 2, a non-GAS, causes STSS is yet to be unveiled.

For the CPS gene clusters, both the 2005 and the 1998 isolates gave rise to a pattern different from S. suis P1/7 ( Figure 5), suggesting specific characteristics of the S. suis 2 isolates from China which could contribute to the high virulence of the bacteria and their ability to cause human STSS. However, additional experiments are needed to examine a possible link between these genotypes and human pathology.

Our detailed analysis of the S. suis 2 isolates allowed a molecular diagnosis in the two outbreaks, and may help to develop some diagnostic products for S. suis 2 in the future.

To our knowledge, this is the first description of a large-scale STSS outbreak among humans caused by a non-GAS pathogen, *S*. *suis* 2. The molecular mechanisms in the pathogen and the host underlying the outbreaks should be addressed in future studies.

## Supporting Information

Alternative Language Abstract Chinese-Language Version of the Abstract, Translated by GFG and YF (25 KB DOC)

Protocol S1 Detailed Materials and Methods (102 KB DOC)

Table S1 Primers for Qualitative PCR Detection (39 KB DOC)

Table S2 Types of Exposure by Patients to Ill/Dead Pigs (27 KB DOC)

**Patient Summary**

**Background**

*Streptococcus suis (S. suis)* is a bacterium that causes disease in pigs. The bacterium is present in nearly all countries with an extensive pig industry, and it can be transmitted between animals and humans. Humans can be infected with S. suis when they have exposed cuts and abrasions on their hands and handle infected pig carcasses and meat. Human infection is rare but can be severe.

**Why Was This Study Done?**

In 2005 there was a serious human disease outbreak in Sichuan province in China that affected more than 100 people who had been working with pigs. It coincided with an outbreak among the local pigs that had the hallmarks of being caused by S. suis bacteria. The human outbreak, however, was unusual: it was larger than any previous ones, many of the human patients died, and the symptoms they had were somewhat different from what had been seen in previous cases of human S. suis infection. This study represents the first detailed scientific report of the human outbreak, including details of the patients and of the bacteria that caused the outbreak. Such information is essential for the global health community to keep tabs on infectious disease outbreaks, and to work together to limit the threat of existing and emerging pathogens.

**What Did the Researchers Do and What Did They Find?**

They collected hospital records and autopsy samples from human patients and from pigs that had died during the outbreak. As they report, 204 human cases were documented during the outbreak, and 38 of those patients died. Many of the infected patients, and almost all of the ones who died, had the typical symptoms of a dangerous condition in which the patient's blood pressure drops (the condition is called Streptococcal toxic shock syndrome, or STSS). Up to the date of this study, STSS had only been documented in patients infected with *Streptococcus pyrogenes,* also a member of the Streptococcus family of bacteria but quite different from S. suis. However, the bacteria that the researchers isolated from the human and pig samples were clearly of the S. suis type, and they showed that the isolated bacteria could cause typical S. suis disease in piglets. The researchers also examined the genetic material of the S. suis from the outbreak to see whether it could explain why these particular bacteria were able to cause STSS. However, they failed to detect any of the genes that are present in S. pyrogenes and thought to cause STSS. They also compared the genetic material from the S. suis outbreak with other S. suis strains from around the world, including one from an earlier, smaller S. suis outbreak in Jiangsu Province that had killed 14 out of 25 patients in the reported human cases. The strains of S. suis that caused the two Chinese outbreaks were more similar to each other than to any other strains from elsewhere in the world.

**What Does This Mean?**

The recent outbreak of a disease transmitted from animals to humans, and in many cases deadly, is of global concern. More experiments are necessary to see whether the size and high death rate of the recent outbreak is because the Chinese S. suis version is a particularly deadly strain, or because of other circumstances specific to this outbreak. Future studies are urgently needed to better understand the Chinese S. suis bacteria in detail. Doctors around the world should be aware of the possibility of S. suis–associated STSS when they see sick, feverish patients who have been in contact with pigs.

**Where Can I Find More Information Online?**

Pages from the UK Health Protection Agency on *Streptococcus suis:* http://www.hpa.org.uk/infections/topics_az/zoonoses/strep_suis/menu.htm

A short description of the 2005 of the S. suis outbreak in pigs on the WHO Web site: http://www.who.int/csr/don/2005_08_03/en/

WHO fact sheet on *S. suis:* http://www.wpro.who.int/media_centre/fact_sheets/fs_20050802.htm

Pages on S. suis from the Hong Kong Centre for Health Protection: http://www.chp.gov.hk/content.asp?langs=en&info_id=3648&id= 24&pid=9

Pages from the Chinese Center for Disease Control and Prevention:

http://www.chinacdc.net.cn/n272562 (English home page) http://www.chinacdc.net.cn/n272442/n272530/index.html (Chinese home page)

## Abbreviations

CPS: capsule polysaccharide
GAS: group A streptococcus
mrp: muramidase release protein
multi-PCR: multiplex PCR
RFLP: restriction fragment length polymorphisms
*S. suis* 2: *Streptococcus suis* serotype 2
SS2: *Streptococcus suis* serotype 2
STSS: streptococcal toxic shock syndrome
TEM: transmission electron microscopy
TSS: toxic shock syndrome
