# Correction: Streptococcal Toxic Shock Syndrome Caused by *Streptococcus suis* Serotype 2

**DOI:** 10.1371/journal.pmed.0030377

**Published:** 2006-08-29

**Authors:** Jiaqi Tang, Changjun Wang, Youjun Feng, Weizhong Yang, Huaidong Song, Zhihai Chen, Hongjie Yu, Xiuzhen Pan, Xiaojun Zhou, Huaru Wang, Bo Wu, Haili Wang, Huamei Zhao, Ying Lin, Jianhua Yue, Zhenqiang Wu, Xiaowei He, Feng Gao, Abdul Hamid Khan, Jian Wang, Guo-Ping Zhao, Yu Wang, Xiaoning Wang, Zhu Chen, George F Gao

In *PLoS Medicine*, volume 3, issue 5: DOI:  10.1371/journal.pmed.0030151

As part of the revision of this manuscript, additional data on the clinical description of the cases were added, as were four new authors who had been involved in this part of the work. Those same four researchers had also authored a related manuscript, which should have been mentioned in the *PLoS Medicine* article. The related manuscript is now published:

Yu H, Jing H, Chen Z, Zheng H, Zhu X, et al. (2006) Human *Streptococcus suis* outbreak, Sichuan, China. Emerg Infect Dis 12: 914–920. Available at http://www.cdc.gov/ncidod/EID/vol12no06/05-1194.htm.

