# Supplementary material for: Streptococcal Toxic Shock Syndrome Caused by Streptococcus suis Serotype 2
Source: PLoS Med. 2006 Apr 11;3(5):e151. doi: 10.1371/journal.pmed.0030151 (PMC1434494; doi:10.1371/journal.pmed.0030151)
Supplement: Supplementary file 1 [file pmed.0030151.sd001.doc]

**Chinese Title: 猪链球菌2型引起的链球菌中毒性休克综合征**

**Chinese Abstract摘要**

**背景**	猪链球菌2型（*S*. *suis* 2, SS2）是一种重要的人畜共患病的病原体，既往报道人类感染主要引起散发型急性脑膜炎和败血症。 截止到目前，由非SS2的A组链球菌（GAS）引起链球菌中毒性休克综合征（STSS）有系统的研究，而其他链球菌所致的TSS鲜见。 然而，近来在中国四川省人群中发生的一次大规模的SS2暴发疫情，呈现高侵袭、深部组织性感染的STSS症状。 其临床主要表现为急起高热、皮下淤血（癍）、低血压、休克和多器官功能衰竭等。

**方法和结果**	针对该次公共卫生事件，我们开展了系统的临床和实验研究。 通过对病人的临床和病理研究，首次揭示了由SS2所致STSS的典型特征，而此前绝大多数STSS均源于GAS链球菌感染。回顾分析显示，本次暴发与1998年在中国江苏省暴发的疫情类同。本次四川SS2暴发疫情分离到37株人源细菌菌株和8株猪源细菌菌株。1998年江苏SS2暴发分离到3株人源细菌菌株和2株猪源细菌菌株。通过流行病学调查、光学显微镜观察、临床特征分析、分离菌对实验猪感染试验、多重PCR检测、限制性片段长度多态性（RFLP）和基因序列比对等手段，对病原体进行了综合鉴定，确认高致病性的SS2是这两次STSS暴发的罪魁祸首。

**结论**	据我们所知，这是首次报道在中国暴发的两起由SS2（一种非A组链球菌）引发的大规模STSS疫情。 2005年暴发中，人感染病例204例，死亡38例；1998年暴发中，人感染病例25例，死亡14例。 其中绝大多数死亡病例临床上均表现为典型的STSS，仅个别死亡病例表现为脑膜炎。 由SS2感染人群所致STSS的分子机制目前还不清楚，值得进一步探讨。
