# Supplementary material for: Streptococcal Toxic Shock Syndrome Caused by Streptococcus suis Serotype 2
Source: PLoS Med. 2006 Apr 11;3(5):e151. doi: 10.1371/journal.pmed.0030151 (PMC1434494; doi:10.1371/journal.pmed.0030151)
Supplement: Supplementary file 2 [file pmed.0030151.sd002.doc]

**Protocol S1. Detailed Materials and Methods**

***Isolation and identification of the pathogen.*** To isolate the possible bacterium pathogen, the following selective media were utilized to propagate the inoculum at 37°C cultivation incubator for 48 hrs [13]: Nutritional broth, L-nutritional broth, alkaline tryptone, MAC agar, SS agar, sorbitol MAC agar, TCBS agar, L-agar, nutritional agar with 8% (vol/vol) of the bovine serum, and nutritional agar with 8% (vol/vol) of the defibrinated sheep blood.

***Light and electron microscopy of the pathological sections.*** The liver and spleen tissues from the autopsy specimens were selected to prepare the pathological sections with the standard method [13]. Briefly, liver and spleen tissues were fixed in 10% (vol/vol) neutral-buffered formalin. After routine tissue processing including dehydration by graded alcohol solutions, washing, and incubation in paraffin, 4 µm thick sections were cut and stained with hematoxylin and eosin (H&E). About 2 hrs later, the prepared tissue sections were observed under the optical microscopy (Olympus, Japan).

The transmission electron microscopy (TEM) was utilized to obtain more detailed pathological information responsible for the major symptom. Similar to the standard protocol [13], the tissue samples were fixed in 2.5% (vol/vol) dialdehyde for 2 hrs, post-fixed in 1% (vol/vol) osmium tetroxide for 1 hr, dehydrated in graded ethanol, and embedded in Epon-812 epoxy resin. Then 70 nm ultra-thin sections were produced and quickly stained in aqueous uranyl acetate and Reynolds’ lead citrate. Finally, the generated tissue sections were examined using a JEM-1200 TEM (Jeol Ltd. Tokyo, Japan).

***Immunohistochemistry experiments.*** To detect the existence of the bacteria/antigens in the infected tissues, the immunohistochemical experiment was performed on formlin-fixed paraffin-embedded liver sections. Briefly, sections were deparaffinized and rehydrated in graded concentrations of ethanol. Endogenous peroxidase activity was blocked with 3% (vol/vol) H_2_O_2_. Sections were placed in 0.010 mol/L citrate buffer (pH6.0), and heated in microwave oven. Non-specific protein binding was inhibited by 40 min incubation in 5% (vol/vol) horse serum. Primary antibody (anti-SS2 serum derived from the recovered pig infected by *S*. *suis* 2, normal serum from the pig unaffected by *S*. *suis* 2, diluted 1:50) was applied. Detection was performed using the Histo-stain Plus Kit (Zymed). Sections were briefly counterstained with haematoxylin.

***Pig infection experiments.*** To test the pathogenicity of the bacterium agent, the experiments were performed according to the method described by Allen et al. [14] with some modification. In summary, one of those human isolates from Sichuan of the 2005 outbreak, termed as *S*. *suis* ZYH, was selected to carry out the infection experiment, in comparison with a mild virulent strain, *S*. *suis* ZY68 (isolated from a healthy pig in China and kept in our laboratory). The sperm-free minipiglets (Guizhou line) aged 44 days were obtained and screened for the presence of major pathogens prior to challenge. The bacteria for challenge were grown overnight in THB at 37°C, and then were sub-cultured on the same condition till the OD_560_ reached to 1.0. Then the cultures were diluted with the sterilized 1×PBS buffer (pH7.3) into 5.0×10^7^ CFU, and used to infect those mini-pigs via intravenous injection or intranasal route under anesthesia.

***Mitogenicity activity test to search for superantigens.*** The mitogenicity of *S*. *suis* 2 was determined using a method as previously described [21, 22]. Briefly, 4 strains of *S*. *suis* 2 from both outbreaks (2 each) were cultivated for collection of the supernatants. Human lymphocytes isolated from the healthy blood, were prepared into peripheral blood mononuclear cells (PBMCs) in RPMI 1640 medium (Gibco). RPMI 1640 medium supplemented with 2% fetal calf serum (Gibco), 100 U/mL of penicillin (Mediatech Cellgro), 100 mg/mL of streptomycin (Mediatech Cellgro), and 2 mM L-glutamine (Mediatech Cellgro) was used to culture human PBMCs. Lymphocytes were then used at a concentration of 2×10^5^/mL cells per well in 200 ml volumes. Serial 10-fold dilutions of supernatants (beginning at 10 mg/well) were added to the wells, and each dilution was detected in quadruplicate; positive or negative control wells received the supernatants from *S*. *pyogenes* or RPMI 1640 medium only. Plates were incubated at 37°C for 72 hrs in 5% CO_2_, and then 1 µCi of [^3^H] thymidine (Amersham) was added to each well. After another 18 hrs, cells were harvested onto fiberglass filters (Whatman), and incorporation of the [^3^H] thymidine was determined using a scintillation counter.

***Preparation of genomic DNA.*** Routine genomic DNA preparations from all the isolates were performed as described by Sambrook et al. [15]. In brief, 1.5 ml of each bacterium cultivation were centrifuged (12,000 rpm, 4°C, 20 min), the pellets were resuspended in a mixture of 567 µl of 10 mM Tris-HCl (pH 8.0), 10 mM EDTA, 30 µl of 20% SDS, and 3 µl of proteinase K (20 mg/ml; Sigma-Aldrich Chimie), and then maintained at 37°C for ~1 hr. 70 µl of 10% (w/v) CTAB, and 0.7 M NaCl were added gently and incubated at 65°C for ~10 min, followed by the mixture of 100 µl of 5 M NaCl (pH 8.0). After being cooled to room temperature, the samples were placed with equal volume of phenol/chloroform/isoamylic alcohol (25:24:1), mixed and centrifuged at 12,600 rpm for 20 min. Then, the supernatants were mixed with 50 µl of 3 M sodium acetate buffer (pH 5.5) and 400 µl of isopropanol for 30 min at 4°C to precipitate the DNA. Finally, the DNA pellets were washed with 70% (v/v) ethanol, dried, and dissolved in 100µl of double-distilled water (ddH_2_O), after the centrifugation at 12,800 rpm for 30 min.

The isolated total DNAs were all separated by electrophoresis with 0.8% agarose gel (Brazil) to determine the quality and density before used as templates of PCR amplifications.

***Qualitative Polymerase chain reaction (PCR) detection.*** Multiplex-PCR assay was developed according to the methodology described by three research groups [16-18] with some modifications. Six pairs of primers specific for the target genes including *16S rDNA,* *gdh*, *mrp*, *epf*, *suilysin*, and *cps-2J*, were designed in this study (listed in Table.1). PCR was conducted in a total volume of 50 µl containing 10 mM Tris-HCl (pH 8.3); 1.5 mM MgCl_2_; 50 mM KCl; 0.001% gelatin (Takara); 200 µM of each deoxynucleoside triphosphate (dATP, dCTP, dGTP, and dTTP) (Takara); 1 pmol of each primer; 2.5 U of ExTaq polymerase (Takara) and ~2 ng of genomic DNA template.

The Multiplex-PCR assay was performed in PTC-225 thermocycler (MJ Research), comprising 5 min of pre-incubation at 95°C, followed by 35 cycles of 0.5min at 94°C, 1.0 min at 55°C, and 3.0 min at 72°C (but the mono-PCR procedure used for both *16S rDNA* and *gdh*, consisted of 30 cycles of denaturation at 95°C for 30 sec, primer annealing at 58°C for 30 sec, and extension at 72°C for 60 sec). Final extension was kept for 8 min at 72°C.

The PCR products were separated in a 1.5% agarose gel electrophoresis consisting of ~ 0.2% ethidium bromide (EB) in 0.5×TBE buffer (90 mM Tris, 90 mM borate, 2.5 mM EDTA (pH 8.0)) for 30 min at a constant voltage of 136 V, and then were visualized by UV trans-illumination (Bio-Rad). The 100 bp DNA Ladder (Ferments) was used as a molecular size standard.

***Multi-alignments of the sequences and generation of the phylogenic trees.*** The 16S rDNA sequences, and the deduced amino acid sequences (GDH, Sulilysin, FBP, MRP, EF, and CPS2 gene clusters) were submitted to the data library of Vector NTI Suite 8.0, artificially adjusted, finally assembled into the complete sequences with the software of Vector NTI Suite 8.0. Subsequently, the comparison of *cps2* gene clusters of the typical isolates, derived from the severely infected swine and humans in Sichuan province, China 2005 and Jiangsu province, China 1998, were conducted with a universal reference strain, a highly pathogenic *S*. *suis* P1/7. Meanwhile, six phylogenetic trees of four isolates (named as ZHS3, ZYS8, ZYH13, and ZYH14) were drawn using MEGA version 3.0 [19] on the basis of the 16S rDNA sequence, and the five putative virulence-associated factors’ amino acid sequences in comparison with other known sequences, respectively.
