# Supplementary material for: Streptococcal Toxic Shock Syndrome Caused by Streptococcus suis Serotype 2
Source: PLoS Med. 2006 Apr 11;3(5):e151. doi: 10.1371/journal.pmed.0030151 (PMC1434494; doi:10.1371/journal.pmed.0030151)
Supplement: Supplementary file 3 [file pmed.0030151.st001.doc]

**Table S1. Primers for qualitative PCR detectio**n

| Gene | Primer sequence | Location | Tm (°C) | Size (bp) |
| --- | --- | --- | --- | --- |
| *16S rDNA* | P1: 5’-taacagtatttaccgcatg-3’ | 168~186 | 58 | 1326 |
|  | P2: 5’-taccttgttacgacttca-3’ | 1494~1477 |  |  |
| *gdh* | P1: 5’-tcaagtcaaccgtggcta-3’ | 534~551 | 56 | 657 |
|  | P2: 5’-tgctgaaaatacgagtgc-3’ | 1190~1173 |  |  |
| *mrp* | P1: 5’-tgctgaaaatacgagtgc-3’ | 392~ 409 | 50 | 970 |
|  | P2: 5’-tgccadcataatcataccc-3’ | 1361~1344 |  |  |
| *epf* | P1: 5’-aaaacggatgaagcgtt-3’ | 2613~2630 | 56 | 744 |
|  | P2: 5’-aattagagctgccattgg-3’ | 3556~3339 |  |  |
| *suilysin* | P1: 5’-ggtgcttadtgttgcgaaa-3’ | 1877~1894 | 48 | 622 |
|  | P2: 5’-gcccafttatgtcattcct-3’ | 2498~2481 |  |  |
| *cps-2J* | P1: 5’-tgatagtgatttgtcgggaggg-3’ | 13909~13929 | 56 | 557 |
|  | P2: 5’-gagtatctaaagaatgcctattg-3’ | 14465~14444 |  |  |
