# Supplementary material for: Streptococcal Toxic Shock Syndrome Caused by Streptococcus suis Serotype 2
Source: PLoS Med. 2006 Apr 11;3(5):e151. doi: 10.1371/journal.pmed.0030151 (PMC1434494; doi:10.1371/journal.pmed.0030151)
Supplement: Supplementary file 4 [file pmed.0030151.st002.doc]

**Table S2.** **Exposure types of patients to ill/dead pigs**

| Types | Number of patients (204) |
| --- | --- |
| Slaughtering | 142 |
| Washing and cutting | 138 |
| Conveying or transportation | 168 |

* The patients consisted of 198 farmers, 5 butchers, and 1 veterinarian. The number of patients with different exposure types was overlapped each other.
